# Sympathetic Chain Ganglia in the Female Pig During Prenatal Development: Noradrenergic and Cholinergic Neurons

**DOI:** 10.3390/cimb48020175

**Published:** 2026-02-03

**Authors:** Amelia Franke-Radowiecka

**Affiliations:** Department of Animal Anatomy, Faculty of Veterinary Medicine, University of Warmia and Mazury in Olsztyn, Oczapowskiego 13, 10-719 Olsztyn, Poland; ameliaf@uwm.edu.pl; Tel. +48-89-523-37-33

**Keywords:** neuronal development, peripheral nervous system, ganglia, neurotransmitters, foetus, immunohistochemistry

## Abstract

Due to the limited data on chemical coding of sympathetic chain ganglia neurons during the prenatal period, this study, for the first time, aimed to characterise noradrenergic and cholinergic neurotransmitter expression in lumbar sympathetic chain ganglia (L SChG) of 5-, 7-, and 10-week-old porcine foetuses as a model increasingly recognised in biomedical research. Double immunohistochemical staining was performed using antibodies against PGP 9.5 (marker of neuronal structures), β-hydroxylase tyrosine (DβH), and vesicular acetylcholine transporter (VAChT). The current findings demonstrated that, in 5-week-old foetuses, approximately 79.83 ± 4.37% of nerve cell bodies were DβH-positive, 25.90 ± 5.60% contained VAChT, and some neurons were DβH/VAChT-positive (12.45 ± 4.36%). In 7-week-old foetuses, the proportion of DβH-positive neurons increased to 82.0 ± 9.7%, while VAChT-positive neurons decreased to 6.5 ± 1.0%, and 9.1 ± 0.7% DβH-positive L SChG perikarya contained VAChT. In 10-week-old foetuses, DβH-positive neurons accounted for 88.5 ± 2.1%, VAChT-positive for 1.98 ± 0.64%, and DβH/VAChT-positive perikarya decreased to 5.4 ± 0.4%. These findings provide new insight into the differentiation of the autonomic nervous system and the timing of neurotransmitter phenotype specification. Understanding the ontogeny of noradrenergic and cholinergic neurons may contribute to a better understanding of developmental disorders affecting the autonomic nervous system and may have implications for regenerative medicine, neurodevelopmental diagnostics, and therapeutic strategies targeting sympathetic dysfunction.

## 1. Introduction

The sympathetic trunk is a paired chain of ganglia (sympathetic chain ganglia, SChG) connected by interganglionic branches and runs bilaterally along the spine from the base of the skull to the coccygeal vertebrae [1,2,3]. SChG is a key component of the autonomic nervous system and helps regulate internal organ function and maintain homeostasis. The neurons of SChG, through their axons, innervate various organs and blood vessels, modulating their functions. The sympathetic part of the autonomic nervous system activates the body in response to stress, preparing it to react to threats. Its activation leads to increased heart rate, bronchodilation, inhibition of gastrointestinal motility, and increased sweat secretion [1,4]. This action is antagonistic to the parasympathetic nervous system, which promotes regenerative and energy-conserving processes. The peripheral autonomic nervous system arises from neural crest cells (NCC), which emerge during neuroectodermal differentiation, between days 17 and 21 in humans and days 13 to 15 in pigs [5]. SChG derive from NCC located at the level of somites 8–28 [6,7]. These cells migrate ventromedially toward the dorsal aorta under the influence of signalling molecules such as Eph/ephrin, Neuropilin/Semaphorin, and F-Spondin [8,9,10]. At the dorsal aorta, NCC lose their segmental organisation, intermingle, and reaggregate into ganglia, a process regulated by N-cadherin and ephrin-B1 [7,11,12]. Semaphorin 3A repels Nrp1-positive NCC, supporting their proper positioning [13], while BMP-4 secreted by the aorta induces differentiation into sympathetic neurons [10,14]. Final segmentation of the ganglia into neuronal clusters and non-neuronal regions marks the completion of sympathetic trunk formation [15]. Recent studies have shown that Schwann cell precursors (SCPs) from motor nerves contribute to the formation of sympathetic neurons and satellite glia, and the removal of these nerves results in mispositioning and fragmentation of the sympathetic chain [4]. Motor nerve-derived Semaphorin (Sema3A, Sema3F) regulate the organisation of sympathoblasts and axonal growth [4]. Abnormal development of SChG resulting from defects in the migration or differentiation of NCC can lead to fragmentation of the ganglionic chain, the presence of ectopic neuronal clusters, and impaired innervation of organs such as the lungs in the case of congenital diaphragmatic hernia [3]. As a consequence, pulmonary hypertension, autonomic deficits in newborns, and long-term metabolic and cardiovascular complications may develop later in life [3,16]. Morphological studies have also shown that the number of ganglia in the sympathetic trunk changes during the prenatal period and is higher than the standard assumed number [17]. Moreover, sympathetic neurons are unique among neurons in continuing to divide after differentiation into neurons, even after the end of foetal life [12]. This underscores the need to expand current knowledge regarding the development of SChG during the later stages of foetal life, as most existing studies focus on the embryonic period and describe the molecular mechanisms underlying gangliogenesis primarily in humans [12,18,19], rodents [10,20,21,22,23], chick embryos [12,20,23], and zebrafish [19,20,24]. The basic neurotransmitter within the SChG is noradrenaline, synthesised in sympathetic neurons through a series of enzymatic reactions comprising the catecholamine biosynthetic pathway. Key enzymes involved in this process include tyrosine hydroxylase (TH) and dopamine β-hydroxylase (DβH), both of which serve not only as catalytic components but also as established neuronal markers for the identification of adrenergic neurons. The adrenergic subpopulation primarily innervates blood vessels and smooth muscles and forms nerve plexuses within internal organs. They influence the activity of vascular smooth muscle (causing vasoconstriction and an increase in arterial blood pressure), modulate cardiac function (by increasing heart rate and contractile force), and inhibit gastrointestinal activity. They also participate in the mobilisation of energy reserves by stimulating glycogenolysis and lipolysis and indirectly contribute to bronchodilation and thermoregulation [25]. In parallel, a sparse population of cholinergic neurons is also present, using acetylcholine as their primary neurotransmitter. A key protein responsible for the transport of acetylcholine into synaptic vesicles and its subsequent release is the vesicular acetylcholine transporter (VAChT), which is essential for cholinergic transmission in certain autonomic pathways. Cholinergic neurons innervate eccrine sweat glands [26], the periosteum [27], and the rat [28] and porcine mammary glands [29,30]. In chickens, cats, and guinea pigs, cholinergic neurons of the sympathetic trunk have also been observed to project to the blood vessels of skeletal muscles, exerting a vasodilatory effect [31]. Finally, a small group of neurons in SChG are so-called non-adrenergic, non-cholinergic (NANC) neurons, which are used as neurotransmitters for nitric oxide, somatostatin, galanin, and/or vasoactive intestinal peptide. These peptides are also co-expressed with classical neurotransmitters and act as neuromodulators. They play essential roles in regulating vascular tone, glandular secretion, pain modulation, and neuronal plasticity, both under physiological and pathological conditions. During prenatal life, the expression of both DβH and VAChT undergoes dynamic changes, reflecting the maturation of neurotransmitter phenotypes in developing sympathetic ganglia. At early developmental stages, a subset of neurons exhibits a bimodal profile, indicating their neurochemical plasticity [12,32]. The available information primarily concerns the embryonic period, with sporadic observations of changes in later stages of prenatal development. These data provide valuable insights; nevertheless, they remain fragmentary, particularly during the foetal period, when organs develop rapidly and current knowledge about the formation of peripheral nervous system structures and the chemical coding of neurons and nerve fibres is very limited. Due to the lack of systematic and quantitative data on the presence of DβH and VAChT in lumbar sympathetic chain ganglia neurons, we decided to analyse the expression of these enzymes at three developmental stages: 5, 7, and 10 weeks of prenatal development in porcine foetuses, a model species widely recognised as critically important in biomedical research [33]. Given the unique and difficult to obtain nature of the material, the present study focuses on these three specific developmental stages, which represent the most consistent and uniform set of samples currently available. This study builds upon previously published research examining the development of the paracervical ganglion and internal reproductive organs in the porcine foetus [34,35,36], as discussed in the following section. Consequently, to maintain group uniformity in the present study and to ensure continuity with previous research, only female foetuses were used. Collectively, these data offer a broader perspective on the changes occurring in developing structures of the peripheral autonomic nervous system at defined developmental time points. The findings may contribute to a better understanding of the processes involved in the formation of ganglia, organ development, organ innervation, and the relationships between these elements.

## 2. Materials and Methods

The porcine foetuses (*Sus scrofa domesticus*) were obtained from a slaughterhouse. According to Polish law and EU Directive No 2010/63/EU, the experiments performed in the present study do not require the approval of the Ethics Committee. In the current study, 5-week-old (*n* = 5; 3–4 cm long), 7-week-old (*n* = 5; 7–8 cm long), and 10-week-old (*n* = 5; 13–14 cm long) foetuses were used. To ensure the uniformity of the study groups, only female foetuses were selected for the study. The age of the foetuses was marked according to the crown-rump length (CRL) method. CRL sets the distance from the top of the head of the embryo or foetus entering the lower limit of the buttocks [34].

### 2.1. Tissue Preparation and Immunohistochemistry

No more than 10 min elapsed between the sow’s slaughter and the beginning of the fixation procedure of the foetuses. After removing the foetuses from the uterus, the abdominal skin was cut for better penetration of the fixative solution in all collected animals. Foetuses were fixed by immersion in 4% buffered paraformaldehyde (pH 7.4; times of incubation of 5-, 7-, and 10-week-old foetuses were 1 h, 2 h, or 4 h, respectively). After fixation, the samples were rinsed with phosphate buffer (pH 7.4; overnight, in a refrigerator). They were then transferred to an 18% buffered sucrose solution (pH 7.4), where they were stored until further processing. Only female foetuses were used in this study. Foetal lumbar backs along with SChG or lumbar SChG ganglia (10-week-old foetuses) were dissected, cut into 10 μm thick cryostat sections (cross or longitudinal section), and processed for single- or double-labelling immunofluorescence as described earlier [36,37,38]. The sections were labelled using antibodies against PGP, DβH, and VAChT, listed in Table 1. In detail, after air-drying at room temperature (RT) for 30 min, the sections were pre-incubated with a blocking mixture containing 10% normal horse serum, 1% bovine serum albumin, and 0.05% Tween 20 in PBS (1 h, RT). After rinsing the sections in PBS (3 × 10 min), they were incubated with a mixture of two heterologous sera containing antibodies against the examined antigens (incubation in a humid chamber, RT, approximately 20 h). The sections were incubated in the following antibody combinations: PGP/DβH (dilution—1:400/1:500), PGP/VAChT (dilution—1:400/1:500), and DβH/VAChT (dilution—1:500/1:1500). After rinsing the sections in PBS (3 × 15 min), they were then incubated with a mixture of appropriate secondary antibodies (1 h, RT, listed in Table 1). After rinsing in PBS (3 × 10 min), the slides were cover-slipped with carbonate-buffered glycerol (pH 8.6). Primary antibodies were not raised against porcine (*Sus scrofa*) antigens. Cross-species applicability was supported by amino-acid sequence conservation analysis using global pairwise alignment (EMBOSS Needle, Needleman-Wunsch, EBLOSUM62). Human and pig PGP9.5 (UCHL1) proteins showed 96.9% identity (216/223 aa); notably, the antibody manufacturer also reports cross-reactivity with pig. Bovine and porcine dopamine β-hydroxylase (DBH) sequences showed 88.7% identity (541/610 aa), consistent with the bovine origin of the immunogen. The VAChT antibody was raised against a rat C-terminal peptide, which showed 84.2% identity (16/19 aa) with the corresponding porcine sequence. The omission of primary antisera and their replacement by normal, non-immune sera (rabbit, mouse, or rat) was used to investigate the specificity of immunohistochemical labelling. No fluorescence was observed in any of these control stainings, which confirmed the specificity of the staining. For preliminary verification of staining quality, a Zeiss Axiophot fluorescence microscope equipped with epifluorescence and an appropriate Alexa Fluor 488 filter was used. Subsequently, the sections were analysed (Alexa Fluor 488 and Alexa Fluor 555 filter, lens Zeiss EC Plan-NEOFLUAR 40×/0.25; 20×/0.8, and 10×/0.3 (Zeiss, Jena, Germany)) and recorded using a Zeiss LSM 700 confocal laser scanning microscope (Zeiss, Jena, Germany) and ZEN Software 2009. Measurements of neuronal cell bodies were also performed using the measurement tools available in this software. The figure set was prepared with CorelDRAW X7 graphical software, version 17.6.0.1021 (Ottawa, ON, Canada).

### 2.2. Counting of Neurons

To determine the percentage of particular neuronal populations (PGP/DβH, PGP/VAChT, DβH/VAChT), in accordance with the previously described method [34], at least 500 PGP-positive perikarya were counted on sections for each of the analysed combinations of antisera (PGP/DβH and PGP/VAChT) in each animal in each age group. For the DβH/VAChT antibody combination, at least 500 DβH-positive perikarya were also counted in the same way as was written earlier. In the developmental stages examined, the L SChG does not exhibit a distinct ganglion structure; rather, its appearance depends on the specific developmental stage analysed. In 5-week-old foetuses, it is presented as a uniform, elongated conglomerate, which, as development progresses, transforms into a heterogeneous segmented structure (10-week-old foetuses) [39]. Due to the large differences in shape, size, and number of ganglia (between studied groups of animals and individual animals in the group), it was decided that sections from the sympathetic trunk located between the last thoracic vertebra (behind the last rib) and the sacrum would be taken for the study. Perikarya were counted manually during microscopical analysis. To avoid double counting of the same neurons, the neurons were counted in every third section. The sections for the study were selected so that they originated from the cranial, middle, and caudal parts of the lumbar segment of the sympathetic trunk. In total, approximately 15 sections (for each combination of antisera) were analysed from each animal. To minimise the subjective influence of the researcher on the results, a blinding method was applied. The experimenter was unaware of the assignment of samples/animals to experimental groups.

Neurons were counted during microscopic observations using a Zeiss LSM 700 confocal laser scanning microscope, equipped with appropriate filter sets, which enabled the visualisation of Alexa Fluor 488 (green) and Alexa Fluor 555 (red) labelling. Only neurons with a visible nucleus were counted on the sections.

All the results are expressed as means ± SD. The number of particular neuronal population was calculated as a percentage of positive neurons to the substances studied out of all perikarya counted, for example, for the PGP/DβH neuronal population: (number of DβH-positive neurons/total number of counted PGP-positive neurons) × 100. IBM SPSS Statistics 28 was used for the statistical analysis. Throughout the study, *p* < 0.05 threshold was adopted as the significance level. The Shapiro–Wilk test was chosen to examine whether the data followed a normal distribution. To investigate differences in quantitative data between groups at different weeks, a group of non-parametric tests was selected (due to the lack of normal distribution in the data), i.e., the Mann–Whitney U test for two groups, and the Kruskal–Wallis test for more than two groups (with the Bonferroni multiple group correction as the post hoc test). For comparisons of results within the studied variables (where assumptions were met), one-way ANOVA with Fisher LSD test and Kruskal–Wallis test were used.

### 2.3. PCR, Sex Identification

Sex identification was determined using the PCR technique [40]. Tail samples from each 5-week-old foetus were collected in PBS, and DNA was then extracted using the modified alkaline lysis method by Lopez 2012 [40] (A Quick, No-Frills Approach to Mouse Genotyping, Manuel E. Lopez, http://www.bio-protocol.org/e244, 5 August 2012). Samples were incubated at 98 °C for 45 min, then flicked and vortexed to release DNA. PCR, performed with Start Warm Master Mix (AA Biotechnology, Gdańsk, Poland) in a Biometra thermocycler, used 1 µL of DNA per reaction. Sex was determined by amplification of the porcine SRY gene (581 bp; primers F: 5′-AAGTCACTCACAGCCCATGAA-3′, R: 5′-CCATGGAAGTTCCTGTATCAT-3′), with GAPDH serving as a quality control (primers F: 5′-ACATTGTCGCCATCAATG-3′, R: 5′-ATGCCCATCACAAACATG-3′). PCR products were resolved on a 2% agarose gel stained with EtBr: SRY band-positive samples were classified as male (A) and band-negative as female (B). Due to the well-developed and visible external genital organs in 7- and 10-week-old porcine foetuses, we decided not to perform additional studies related to sex identification.

## 3. Results

### 3.1. Five-Week-Old Foetus

**Figure 1:**
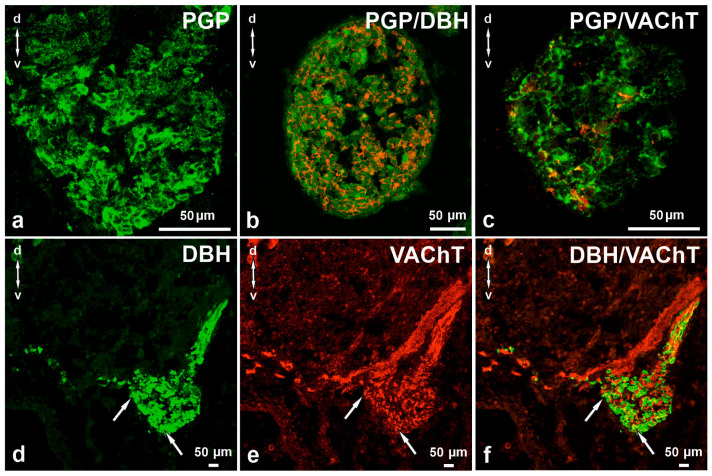
Cross-section of lumbar SChG ganglion in 5-week-old pig foetus. Protein gene product 9.5 (PGP), as a pan-neuronal marker, highlights neural structures. In the ganglion, neurons with large nuclei and a small amount of cytoplasm are visible. The ganglion is characterised by a loose structure (**a**). Many PGP-positive neurons (green) also contain DBH (red, (**b**)). At this stage of foetal development, the presence of VAChT (red) was also observed in many PGP-positive perikarya (green (**c**)). Section showing the L SChG immunolabeled for dopamine-β-hydroksylase (DβH; (**d**)) and vesicular acetylcholine transporter (VAChT; (**e**)). Some neurons contain both studied substances ((**d**–**f**), arrows). (**f**) Merged images (**d**,**e**). d–v—dorsal-ventral orientation. Scale bars = 50 µm.

At this stage of prenatal development, double immunohistochemical staining showed that PGP-positive neurons contained DβH- and/or VAChT (Table 2, Figure 1a–c). Approximately 79.41 ± 0.98% of nerve cell bodies were DβH-positive and 25.90 ± 1.01% contained VAChT. Some neurons were DβH/VAChT-positive (12.45 ± 0.90%).

Perikarya containing DβH were typically intensely stained (Figure 1d,e). DβH-positive neurons with medium to low staining intensity usually also contained VAChT (Figure 1e,f). The size of DβH/VAChT-positive nerve cell bodies was 7–8 μm DβH- and VAChT-positive neurons were distributed evenly throughout the ganglion. In the ganglia, a large number of delicate VAChT- and fewer DβH-positive nerve fibres were observed. A bundle of DβH-, DβH/VAChT-, or VAChT-positive nerve fibres with visible nerve cell bodies running ventrally from the spinal cord to SChG was also occasionally observed. Some of these fibres run toward the ganglia, and some of the fibres then leave the ganglion and fuse with the VAChT-positive, separate bundle of nerve fibres and run ventrally toward the body cavity (Figure 1d,e). Neurons that were observed in VAChT-positive bundles mainly contained VAChT.

### 3.2. Seven-Week-Old Foetuses

**Figure 2:**
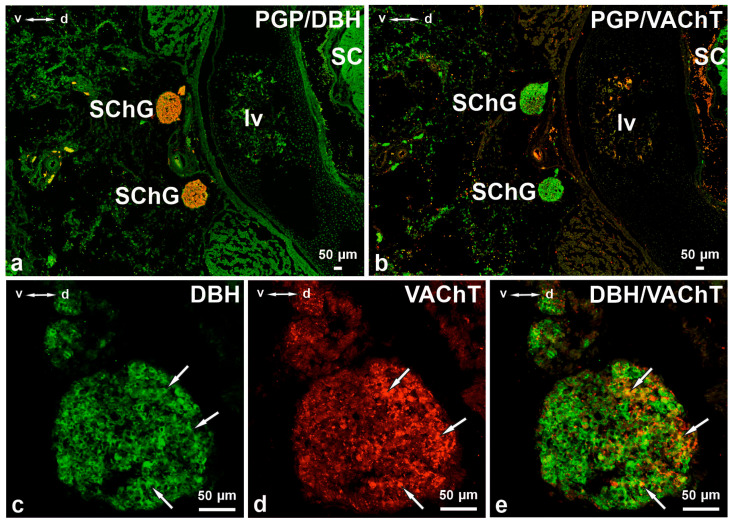
Cross-section of the lumbar region of a 7-week-old porcine foetus back with visible sympathetic chain ganglia. L SChG, as a paired structure, was arranged dorsolaterally from the descending aorta ((**a**,**b**); consecutive section). PGP-positive (green) L SChG neurons contained DβH ((**a**), red) and VAChT ((**b**), red). Mainly middle- or low-intensity DBH-positive cells ((**c**), arrows) contained VAChT (**d**, arrows). DβH- and VAChT-positive nerve cell bodies were distributed evenly throughout the ganglion, but most VAChT-positive neurons were observed in the dorsal part of the ganglion (**c**–**e**). SC—spinal cord, lv—lumbar vertebrae. (**e**) Merged images (**c**,**d**). d–v dorsal-ventral orientation. Scale bars = 50 µm.

**Figure 3:**
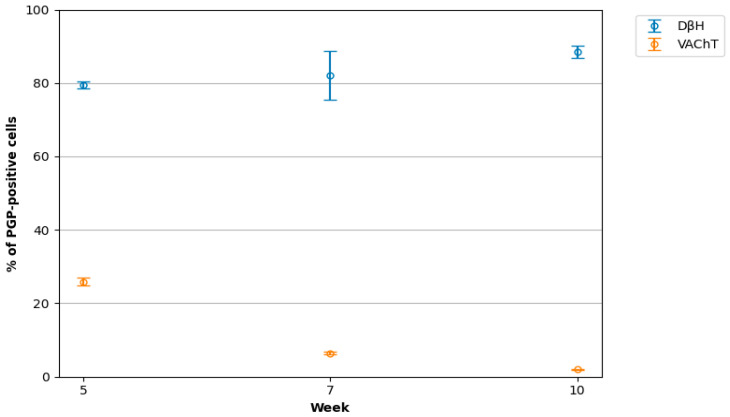
The number of PGP/DβH- or PGP/VAChT-positive neurons in the lumbar sympathetic chain ganglia (L SChG) of 5-, 7-, and 10-week-old porcine foetuses. This study demonstrated a progressive increase in the proportion of DβH-positive neurons within the population of PGP-positive neurons during prenatal development, with mean values rising from week 5 to week 7 (+2.60%) and further to week 10 (+9.11% compared to week 5). In contrast, the proportion of VAChT-positive neurons showed a marked decrease over the same developmental period, declining from week 5 to week 7 (−19.45%) and reaching the lowest values at week 10 (−23.92% compared to week 5). Overall differences between age groups were statistically significant for both markers (Kruskal–Wallis test, *p* < 0.001). Pairwise post hoc comparisons confirmed these differences after Bonferroni correction (*p* < 0.001).

**Figure 4:**
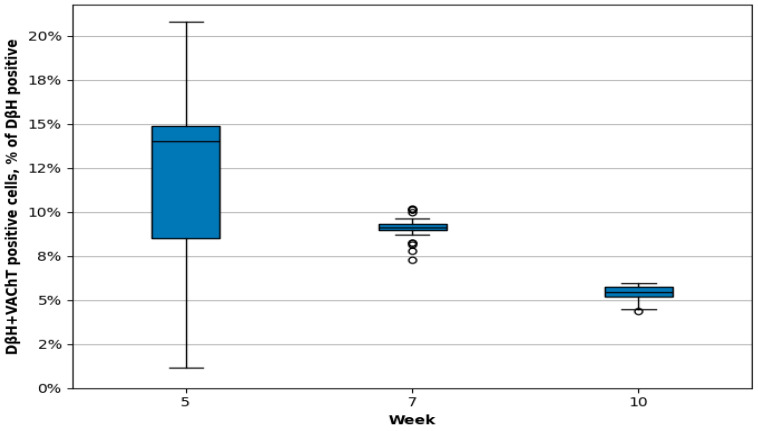
The chart illustrates the percentage of DβH-positive neurons co-expressing VAChT in the lumbar sympathetic chain ganglia (L SChG) of 5-, 7-, and 10-week-old porcine foetuses. Significant differences were observed in the proportion of DβH-positive neurons simultaneously containing VAChT across the developmental stages (Kruskal–Wallis test, *p* < 0.001). Post hoc pairwise comparisons revealed statistically significant differences between all age groups: 5- vs. 7-week-old foetuses (Bonferroni post hoc correction, *p* = 0.012), 5- vs. 10-week-old foetuses (Bonferroni post hoc correction, *p* < 0.001), and 7- vs. 10-week-old foetuses (Bonferroni post hoc correction, *p* < 0.001). The horizontal line represents the median, while the height of the box corresponds to the interquartile range (the difference between the third and first quartile), encompassing 50% of the data. The whiskers extend to 1.5 times the interquartile range, and outliers are indicated by dots.

In 7-week-old foetuses, 82.01 ± 6.65% of PGP-positive L SChG neurons contained DβH and 6.45 ± 0.26% of these neurons contained VAChT (Table 2, Figure 2a–e, Figure 3 and Figure 4). Among the DβH-positive perikarya, 9.08 ± 0.24% also contained VAChT (Table 1). Mainly middle- or low-intensity DβH-positive cells contained VAChT (Figure 2c–e). DβH- and/or VAChT-positive nerve cell bodies were distributed evenly throughout the ganglion. However, most VAChT-positive neurons were observed in the dorsal part of the ganglia (Figure 2d). DβH/VAChT-positive neurons often formed clusters (several to a dozen nerve cell bodies) that were most frequently located closer to the edge of the ganglion rather than its centre. The size of these neurons was 8–12 μm. Many DβH-positive nerve fibres were visible in the whole ganglion. Most of them were observed around intensely and moderately stained DβH-positive pericarya. A few nerve fibres surrounded weakly immunoreactive, DβH-positive nerve body cells. A large number of VAChT-positive nerve fibres were visible in the whole ganglion. These fibres often surrounded VAChT- and DBH/VAChT-positive nerve cell bodies (Figure 2c–e).

### 3.3. Ten-Week-Old Foetuses

**Figure 5:**
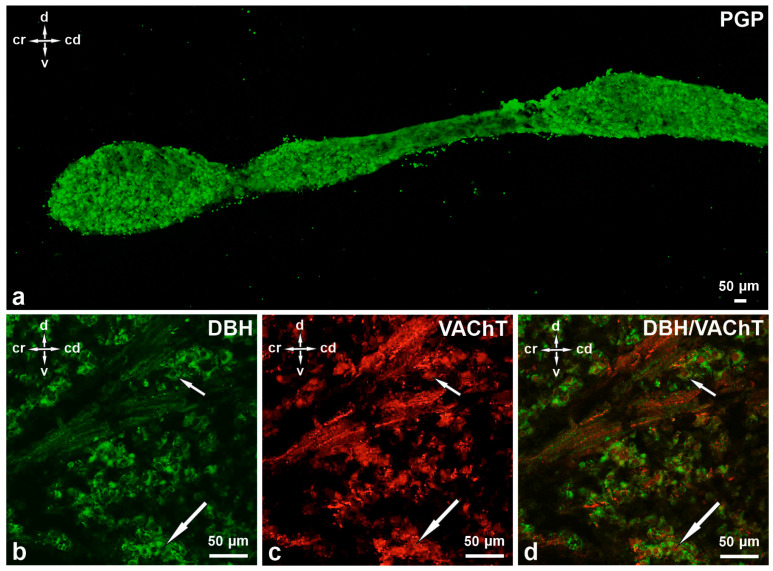
A longitudinal section of the L SChG in a 10-week-old pig foetus with visible differences in the length, width, and shape of the three ganglia ((**a**), PGP staining). Usually, middle- or low-intensity DβH-positive neurons ((**b**), short arrow) contained VAChT ((**c**), short arrow) and were distributed evenly throughout the ganglion. Long arrow—DβH-negative but VAChT-positive neuron. Short arrow—DβH/VAChT-positive neuron (**b**,**c**). Fewer VAChT-positive fibres were observed in the ganglion during the studied period (**c**). They were most frequently located near VAChT- and DβH/VAChT-positive perikarya or observed in bundles running through the ganglion. (**d**) Merged images (**b**,**c**). d-v—dorsal-ventral orientation, cr-cd—cranial–caudal orientation. Scale bars = 50 µm.

At this developmental stage, lumbar SChG neurons contained DβH (88.52 ± 1.65%) and 1.98 ± 0.20% VAChT. In total, 5.38 ± 0.0.18% of DβH-positive neurons also contained VAChT (Table 2, Figure 3, Figure 4 and Figure 5a–d). Many middle- or low-intensity DβH-positive neurons contained VAChT and were distributed evenly throughout the ganglion. The size of these nerve cell bodies was 11–15 µm. A large number of DβH-positive nerve fibres were observed in the whole ganglion, and these fibres had moderate staining intensity (Figure 5b). Intensely stained DBH-positive fibres were mainly found near the intensely stained DβH-positive perikarya. Fewer VAChT-positive fibres were observed in the ganglion in the 10th week of prenatal development (Figure 5c). They were most frequently located near VAChT- and DβH/VAChT-positive perikarya or observed in bundles running through the ganglion (Figure 5b–d).

## 4. Discussion

A previous study dealing with the chemical coding of SChG neurons revealed that about 95% of them express catecholamine-synthesising enzymes such as tyrosine hydroxylase (TH) and DβH in adult mammals [32,41,42,43]. For VAChT-positive neurons, this population is estimated at 3–5% [32,40]. The neurochemical coding scheme of individual SChG mature neurons is related to the target tissue type [26,43]. However, neurons may also differ (depending on the tissue innervated) in soma size, cell membrane properties, dendrite length, and most likely many other functional parameters [26,44]. In different species, including adult pigs [26], adrenergic neurons typically have cell bodies ranging in size from approximately 15 to 60 μm, with the most commonly observed diameter falling between 30 and 50 μm. For example, in the superior cervical ganglion of the rat, these neurons most frequently measure between 35 and 50 μm in diameter [2,45]. Cholinergic neurons are significantly smaller, typically measuring around 20–35 μm [46], and similar values have been reported in the cat [47,48] and rabbit [49]. In interpreting the results of the present study, it should be emphasised that DBH and VAChT immunoreactivity was observed exclusively in PGP-positive cells, and no PGP-negative cells containing these markers were detected. This observation is consistent with previous reports [2,27,47,49] showing that catecholaminergic and cholinergic programmes are activated only after cells commit to the neuronal lineage, whereas non-neuronal cells, including glial and precursor populations, do not express these markers at detectable levels [11,27]. Accordingly, we confidently counted and analysed only perikarya located in the foetal lumbar sympathetic chain ganglia during the examined prenatal periods. The current findings demonstrated that in 5-week-old foetuses, 79.41 ± 0.98% of L SChG neurons expressed DβH, 25.90 ± 1.01% were VAChT-positive, and 12.45 ± 0.90% of DβH-positive neurons co-expressed VAChT. DβH/VAChT-positive neurons measured 7–8 μm, with most neurons in the ganglion ranging from 6 to 8 μm [39]. At seven weeks, the proportion of DβH-positive neurons increased to 82.01 ± 6.65%, while VAChT-positive neurons decreased to 6.45 ± 0.26%, and co-expression of DβH and VAChT was 9.08 ± 0.24%. DβH/VAChT-positive neurons measured 8–12 μm, with most neurons between 8 and 11 μm. In 10-week-old foetuses, DβH-positive neurons accounted for 88.52 ± 1.65%, VAChT-positive for 1.98 ± 0.20%, and DβH/VAChT-positive perikarya decreased to 5.38 ± 0.18%. DβH/VAChT-positive neurons measured 11–15 μm, with most nerve cell body ranging from 12 to 16 μm in this study stage [39]. These results indicate that the most distinct changes in neuronal coding, concerning basic neurotransmitters, occurred between the 5th and 7th weeks of prenatal development. During this interval, a clear consolidation of the noradrenergic phenotype in SChG neurons becomes evident, as demonstrated by the sharp decline in VAChT-positive neurons and the reduction in the population exhibiting the mixed DβH/VAChT phenotype. This transition may be associated with the phase of rapid target tissue development (initiating around the 5th week of foetal development), during which retrograde signals, including various growth factors and cytokines, are released [12]. A developmental pattern has been described in which Bone Morphogenetic Protein (BMP) nearly simultaneously induces both phenotypes and is transiently co-expressed at early stages [50,51,52]. Both sets of neurotransmitter-marker genes may be expressed before target innervation, and through a process of target-dependent selection, the appropriate phenotype is subsequently reinforced and stabilised as the target-specific neurotransmitter profile [27,53,54,55]. With the emergence of the first functional pre- and postganglionic connections, the noradrenergic phenotype becomes dominant, which is reflected in the increased number and intensity of DβH-positive fibres observed at the 7th week of development [56]. The small population of VAChT-positive neurons that persists may represent a subset that later differentiates into specialised cholinergic subtypes described in the adult sympathetic nervous system, including those innervating sweat glands or certain blood vessels [32,57]. Between the 7th and 10th weeks, neuronal coding continued to evolve, with a particularly noticeable decrease in the number of VAChT-positive neurons. Earlier observations of the development of the pelvic plexus and the innervation of the reproductive organs in porcine foetuses revealed that the intensive development of the peripheral nervous system most likely begins around the fifth week of prenatal development. At this time, developing neurons migrate ventrally from SChG towards the body cavity, but the target destination is difficult to predict [36,58]. At this stage, paramesonephric ducts (Müllerian ducts) and genital ridges, from which the ovaries develop, are visible. PGP-positive fibres were observed running along these structures but did not penetrate inside. By the 7th week of development, primary ovaries were already present, and the mesonephric ducts were divided into the tubal and uterine segments as well as the uterovaginal canal [38]. At the level of the uterovaginal canal, neuronal clusters (developing paracervical ganglion) and nerve fibres were observed from which some nerve fibres penetrate the mesenchyme of the paramesonephric ducts [36,38]. Considering that L SChG neurons innervate the organs of the urogenital system, this suggests that around the fifth week of prenatal development in the pig, intensive changes may also occur in the lumbar sympathetic chain ganglia, which involve ganglionic remodelling, the migration and proliferation of immature neurons, and axonal elongation, and their progress through the next weeks of development. This fact may also explain the large number of VAChT-positive (25.90 ± 1.01%) and DβH/VAChT-positive (12.45 ± 0.90%) neurons in the L SChG of 5-week-old foetuses, indicating that they are neurochemically undetermined at this stage of prenatal development. Additionally, when analysing the increase in neuron size between the 5th and 7th weeks of development in the L SChG, it was found that neurons containing both neurotransmitters belong to the group of medium-sized neurons with a tendency to be larger in size in each of the developmental stages studied. This also indicates that their projections are undergoing intensive growth and are extending toward their targets [32]. Studies on the central nervous system have shown that changes in nerve cell body size are correlated with the growth of dendrites and axons, as well as with synaptic processes and neuronal plasticity. Growth factors such as NGF (nerve growth factor) and BDNF (brain-derived neurotrophic factor) stimulate both the enlargement of the neuronal soma and the growth of neuronal projections [32,59]. Furthermore, studies conducted in mice have demonstrated that the projections of more than 50% of DβH/VAChT-positive neurons have not yet reached their targets, indicating that they are still in the growth phase [27,60,61]. Moreover, studies conducted on rodents and chickens have shown that, at early stages of prenatal development, many immature neurons may express both markers [27,60,61]. The expression is induced by inductive signals such as BMP, likely with the involvement of Phox2b. At later stages of development, the expression of cholinergic and adrenergic traits gradually segregates into distinct neuronal populations (adrenergic or cholinergic), which is associated with target selection, when the axons of these neurons reach their destination, and their characteristics are determined by environmental cues [27,62,63,64]. Cholinergic sympathetic neurons represent a specialised subpopulation of the sympathetic nervous system whose neurotransmitter phenotype is secondarily induced by signals derived from target tissues [2,27]. For example, in the urinary bladder, they modulate the tone of the bladder neck and the internal urethral sphincter, whereas in blood vessels, they stimulate endothelial nitric oxide (NO) production via activation of muscarinic receptors on endothelial cells, leading to vasodilation. Studies have demonstrated that these fibres are axons of sympathetic ganglion neurons which, despite expressing cholinergic markers such as choline acetyltransferase (ChAT) and the VAChT, retain characteristic developmental features of sympathetic neurons, including expression of the transcription factor Phox2b. During development, sympathetic neurons are initially noradrenergic by default; however, upon reaching specific target tissues, such as sweat glands, blood vessels, or the urinary bladder, they receive retrograde signals—including ciliary neurotrophic factor (CNTF), leukaemia inhibitory factor (LIF), and gp130 family cytokines—which suppress the adrenergic program and induce expression of ChAT and VAChT [27,32,65]. Importantly, the overall circuit topology, ganglion location, and axonal projection patterns remain unchanged. This process can therefore be described as functional reprogramming without alteration of developmental identity. Consequently, the final phenotype of sympathetic neurons (adrenergic or cholinergic) is determined by signals originating from the target tissue and conveyed retrogradely, encompassing a broad range of growth factors and cytokines. Thus, cholinergic sympathetic neurons do not represent a hybrid or transitional form between autonomic divisions, but rather a fully differentiated sympathetic subpopulation whose neurotransmitter phenotype is secondarily specified by target-derived cues [12].

On the other hand, intensive development of neural structures in the body cavity, such as the pelvic plexus or the extrinsic innervation of the gut [53,66], or of the organs themselves, suggests that while some neurons reach their target, others undergo apoptosis as “unnecessary/in excess”. This may explain the significant decline in DβH/VAChT- and VAChT-positive neurons of L SChG by week 7, whose axons potentially reached their targets, such as the developing internal reproductive organs [36]. From another perspective, a slight increase in the percentage of DβH-positive SChG neurons (from 79.83 ± 4.37% to 82.01 ± 9.67) may also explain the reduction in the number of VAChT-positive neurons, which lose their cholinergic phenotype in favour of an adrenergic phenotype, a process also associated with the massive outgrowth of their axons as they reach their targets [63,67]. In mice, it has been shown that after E14, most sympathetic neurons lose ChAT and VAChT expression due to the influence of local cues and growth factors released by intermediate or final targets that do not support the cholinergic phenotype. This does not necessarily imply a complete loss of the cholinergic phenotype. VAChT-positive neurons may either remain purely cholinergic or exist in a bimodal state, indicating that the adrenergic phenotype is still present but suppressed. Depending on their final target, the definitive neurotransmitter phenotype may only become fully established postnatally [62]. In mouse foetuses, neuronal phenotypes begin to segregate around E13–E14—the majority become noradrenergic, while only selected neurons retain or acquire a cholinergic phenotype, e.g., those that will innervate sweat glands [27]. Between the 7th and 10th weeks of foetal development, the number of DβH-positive neurons increased to 88.52 ± 2.06%, representing an approximate 6% rise. During this period, the number of VAChT-positive neurons decreased to 1.96 ± 0.64%, which is even lower than in adult individuals. Moreover, between the 7th and 10th weeks of development, a significantly greater number of nerve fibres was observed in the L SChG ganglia compared to the previously examined time interval. Based on the previous considerations, it can be assumed that many of the projections of L SChG neurons have reached their targets. As a result, the neurons lose their bimodal character in favour of an adrenergic phenotype. This indicates continued intensive development, likely involving the expansion of interneuronal connection networks and concurrent reorganisation within the structure of the L SChG, adapted to the pace of neural structure development in the body cavity and the maturation of their target organs. It was also often observed that DβH/VAChT-positive neurons exhibited weak to moderate DβH immunoreactivity for the given developmental stage. It is difficult to definitively state that these neurons are undergoing a neurotransmitter phenotype switch, although the direction of this change remains uncertain. It remains unclear whether the adrenergic phenotype is being downregulated through suppression of catecholamine synthesis caused by reduced production of the tyrosine hydroxylase cofactor tetrahydrobiopterin [68], or whether it is being reinforced by a progressive increase in BMP concentration—an environmental cue that becomes available upon axonal target contact [69]. However, the answer may be found in future studies.

Our results, showing dynamic changes in the proportion of DβH- and/or VAChT-positive neurons in the developing lumbar sympathetic chain ganglia (L SChG) of foetal pigs, indicate the existence of a critical period of phenotypic plasticity, the disruption of which may lead to permanent deficits in innervation observed in neurocristopathies [70]. In Hirschsprung’s disease, defects in neural crest cell migration result not only in aganglionosis but also in the loss of sympathetic fibres [70,71,72,73]. In congenital central hypoventilation syndrome (CCHS), caused by PHOX2B mutations, diffuse deficits in autonomic innervation are observed, affecting, among others, the intestines, heart, and respiratory system [68,70,72,74]. In neuroblastoma, immature sympathetic cells fail to consolidate the noradrenergic phenotype, leading to the absence of functional DβH-positive fibres [75,76]. The transient expression of DβH/VAChT observed in L SChG neurons of foetal pigs may correspond to a period of highest vulnerability to disruptions of genes regulating autonomic differentiation (PHOX2B, RET, SOX10). Moreover, the changes observed in the present study suggest that the dynamic maturation of sympathetic ganglia is closely linked to target organ development, as previously observed, for example, in the development of reproductive organs in this species [34,35,36]. Therefore, the porcine model may serve as a valuable tool for developing diagnostic markers of early defects in sympathetic maturation, as well as for creating functional assays of autonomic neuron differentiation in human in vitro systems. The results obtained in this study, based on a unique collection of foetal material, provide an important foundation for further investigations into the prenatal maturation of sympathetic ganglia. The analysed developmental stages demonstrate that this period is characterised by intense and dynamic neurochemical processes, and despite growing knowledge of early embryogenesis, information on the development of the peripheral nervous system during foetal life remains limited. In the future, extending the analysis to additional time points and a broader panel of markers would allow for a more precise determination of the timing of neuronal phenotype transitions. Complementary functional studies and apoptosis analyses could further clarify the mechanisms underlying the maturation and reorganisation of sympathetic ganglia, constituting a natural continuation of the framework established by the present work in the pig.

## 5. Conclusions

This study provides the first comprehensive, stage-specific characterisation of neurochemical differentiation in the L SChG during three different prenatal stages. The dynamic changes observed in the expression of DβH and VAChT between the 5th and 10th weeks of prenatal development indicate a progressive consolidation of the adrenergic phenotype, accompanied by a marked decline in the number of cholinergic (VAChT-positive) neurons. Notably, the presence of DβH/VAChT-positive neurons suggests that some neurons may undergo neurotransmitter phenotype switching or exist in a transitional, bimodal state. These findings support the concept of neurochemical plasticity during autonomic nervous system development and early synaptic organisation. These results fill a significant gap in the understanding of the timing and patterning of neurochemical coding in large mammals and provide a valuable reference for comparative and translational studies of autonomic nervous system development. Importantly, the data were obtained from the pig, a species increasingly and seriously considered in biomedical research due to its anatomical, physiological, and developmental similarities to humans.

## Figures and Tables

**Table 1:** List of primary and secondary reagents used in the study.

***Primary Antibodies***					
**Antigen**	**Clonality**	**Host**	**Dilution**	**Company**	**Catalog No**
Protein gene Product 9.5	monoclonal	mouse	1:400	Biorad (Hercules, CA, USA)	7863-2004
Dopamine beta hydroxylase	monoclonal	mouse	1:500	Millipore (Billerica, MA, USA)	MAB308
Dopamine beta hydroxylase	polyclonal	rabbit	1:500	Enzo (Farmingdale, NY, USA)	BML-DZ1020-0050
Vesicular Acetylcholine Transporter	polyclonal	rabbit	1:5000	Sigma (St. Louis, MO, USA)	V5387
***Secondary Reagents***					
**Antigen**	**Fluorophore**	**Host**	**Dilution**	**Company**	**Catalog No**
Mouse IgG	Alexa 488	goat	1:1000	Invitrogen (Carlsbad, CA, USA)	A-11001
Rabbit IgG	Alexa 555	goat	1:1000	Invitrogen	A-21428

**Table 2:** Percentage of neurons containing the substances studied (DβH, VAChT) in the population of PGP-positive neurons in 5-, 7-, and 10-week-old porcine foetuses L SChG. Numbers displayed as mean ± SD; H-statistic and *p*-value for the Kruskal–Wallis test.

	Week	Mean ± SD	Median	Min	Max	H-Statistic	*p*-Value
PGP/DβH [%] (N = 5)	5	79.41 ± 0.98	80.76	71.09	87.25	10.22	<0.001
	7	82.01 ± 6.65	85.12	37.50	89.19		
	10	88.52 ± 1.65	88.91	78.19	90.73		
PGP/VAChT [%] (N = 5)	5	25.90 ± 1.01	26.19	13.60	36.36	12.50	<0.001
	7	6.45 ± 0.26	6.18	4.30	9.09		
	10	1.98 ± 0.20	1.68	1.03	3.46		
DβH/VAChT [%] (N = 5)	5	12.45 ± 0.90	13.52	1.14	20.78	12.50	<0.001
	7	9.08 ± 0.24	9.13	7.29	10.17		
	10	5.38 ± 0.18	5.44	4.35	5.95		

## Data Availability

The original contributions presented in this study are included in the article. Further inquiries can be directed to the corresponding author.
